# An extension to the OVH concept for knowledge‐based dose volume histogram prediction in lung tumor volumetric‐modulated arc therapy

**DOI:** 10.1002/acm2.70090

**Published:** 2025-04-03

**Authors:** Johann Brand, Juliane Szkitsak, Oliver J. Ott, Christoph Bert, Stefan Speer

**Affiliations:** ^1^ Department of Radiation Oncology Universitätsklinikum Erlangen Friedrich‐Alexander‐Universität Erlangen‐Nürnberg (FAU) Erlangen Germany; ^2^ Comprehensive Cancer Center Erlangen‐EMN (CCC ER‐EMN) Erlangen Germany; ^3^ Comprehensive Cancer Center Alliance WERA (CCC WERA) Erlangen Germany; ^4^ Bavarian Cancer Research Center (BZKF) Erlangen Germany

**Keywords:** DVH prediction, lung tumor, VMAT

## Abstract

**Purpose:**

Volumetric‐modulated arc therapy (VMAT) treatment planning allows a compromise between a sufficient coverage of the planning target volume (PTV) and a simultaneous sparing of organs‐at‐risk (OARs). Particularly in the case of lung tumors, deciding whether it is possible or worth spending more time on further improvements of a treatment plan is difficult. Therefore, this work aims to develop a knowledge‐based, structure‐dependent, automated dose volume histogram (DVH) prediction module for lung tumors.

**Methods:**

The module is based on comparing geometric relationships between the PTV and the surrounding OARs. Therefore, treatment plan and structure data of 106 lung cancer cases, each treated in 28 fractions and 180 cGy/fx, were collected. To access the spatial information, a two‐dimensional metric named overlap volume histogram (OVH) was used. Due to the rotational symmetry of the OVH and the typically coplanar setup of the VMAT technique, OVH is complemented by the so‐called overlap‐*z*‐histogram (OZH). A set of achievable DVHs is predicted by identifying plans in the database with similar OVH and OZH. By splitting the dataset into a test set of 22 patients and a training set of 84 patients, the prediction capability of the OVH‐OZH combination was evaluated. For comparison between the predicted and achieved DVH curves the coefficient of determination *R* ^2^ was calculated.

**Results:**

The total lung showed strong linearity between predicted and achieved DVH curves for the OVH‐OZH combination, resulting in a $R^{2}$ value close to 1 (0.975 ± 0.022). The heart benefits the most of the OZH resulting in a high prediction capability, with a higher $R^{2}$ of 0.962 ± 0.036 compared to the prediction with OVH only (0.897 ± 0.087).

**Conclusion:**

The combination of OZH and OVH was suitable for building a knowledge‐based automated DVH prediction module. Implementing this method into the clinical workflow of treatment planning will contribute to advancing the quality of VMAT plans.

## 1 INTRODUCTION

Treatment planning in radiotherapy, including volume modulated arc therapy (VMAT), involves balancing adequate coverage of the planning target volume (PTV) while simultaneously sparing of the organs at risk (OARs). However, the quality of the treatment planning depends not only on patient specific factors including the type and stage of cancer, the location, and size of PTV and OARs, but also on the planner's level of experience, preferences, and the amount of time the planner is able to invest in the plan.^1^, ^2^, ^3^, ^4^ Hence, in clinical routine, planners often have to handle plans, which fulfil certain criteria, but have the potential of further sparing of the surrounding OARs. Especially in the case of lung tumors with their great variability in PTV size, position and overlap of PTV and surrounding OARs it is difficult to decide whether it is possible or worth spending more time to further spare surrounding organs. In addition to complying with clinic‐specific sparing goals, the planner can ensure plan quality by analyzing DVH curves. The issue, however, is a generalization of the patient that does not take variables such as tumor size or other geometric information into account. Disregarding metrics such as the distance between target volume and OARs may result in either DVH planning objectives that cannot be met or, conversely, objectives that could easily be exceeded and are too loose.^5^ This description leads to a desire for a patient‐dependent metric to predict DVHs. Lately, many methods for DVH prediction have been developed, that is, statistical methods,^6^, ^7^, ^8^ data‐driven strategies^5^, ^9^, ^10^, ^11^, ^12^, ^13^, ^14^, ^15^ incorporating geometric correlations.^5^, ^11^, ^12^, ^16^ Furthermore, based on multi‐criteria optimization and machine learning, methods have been developed that are capable of predicting not only DVHs but also the complete 3D dose distribution. Although these methods are now also available for lung tumors,^17^, ^18^ this work focuses on adapting and further improving the prediction quality of DVHs of OARs using the metric of the overlap volume histogram (OVH) reported by Wu et al.^19^, ^20^ A key advantage of this metric is that it places fewer demands on hardware resources, making it more accessible and easier to implement in various clinical settings.

## 2 METHODS

This research develops a database‐driven model for predicting DVHs for new patients before the actual treatment planning. By utilizing CT scans and associated structure sets, the model compares new treatment plans with existing plans in the database. It then identifies the best‐matching plans and uses them to predict the DVH curves for the new patient. The model is based on comparing geometric relationships between the PTV and the surrounding OARs of a new patient plan based on prior patient plans stored in a database. If there is consistent target coverage among patients, the relative spatial arrangement of an OAR in relation to a target significantly influences the dose distribution received by the OAR. OARs located far from the target are easier to spare, whereas OARs that are proximal or overlapping with the target pose greater difficulty. Hence, the overlap volume seems to be a suitable measure for the prediction of the sparing capability of OARs.^11^ This is especially true if there is a measurable overlap between OAR and the target. The model reaches its limits when the PTV and the OAR are further away from each other and there is little or no overlap. Here, the overlap volume loses most of its significance. In order to continue using the overlap as a measured variable and to include the distance between OAR and PTV, the overlap volume histogram was introduced by Wu et al.^19^ The OVH is a one‐dimensional function that allows a prediction of the DVH for an OAR by comparing it with similar OVHs of other patients. However, even this model has its limits. Due to the rotational symmetry of the OVH and the coplanarity of typical VMAT treatment planning for lung tumors in our clinic, the OVH cannot appropriately describe the spatial correlation of PTV and OAR in cranio‐caudal direction. Hence, it is complemented by a so‐called overlap‐z‐histogram (OZH) which quantifies the overlap of PTV and the corresponding OAR in that direction.

### 2.1 Definition of the OVH

The OVH introduced by Wu et al.^19^ represents the fractional volume of an OAR within a specific distance of a PTV. For a given target T and organ O, the OVH is a one‐dimensional function dependent on the distance r from the PTV and can be calculated by Equation (1)

(1) $$\mathrm{OVH}\left(r\right)=\frac{{∥\left\{p\in O\left|d\left(p,T\right)\leq r\right.\right\}∥}_{V}}{∥{O∥}_{V}}$$

Here, d(p,T) represents the Euclidian distance between a point p within the organ and the boundary of the target. The norm $∥x{∥}_{V}$ is defined as the volume represented by the points of the object in between. Since the volume of the numerator, associated with a fraction of O is always smaller or equal to the volume of the entire organ O in the denominator, the OVH yields a percentage of the OAR's volume. In simpler terms, the OVH quantifies the percentage of the OAR's volume that intersects with a uniformly expanded or contracted target.^19^

### 2.2 Definition of the OZH

The OZH is similar to the OVH and represents the fractional length overlap in cranio‐caudal direction of an OAR within a specific distance of the PTV. It is derived from the orthogonal projections $O^{\prime }$ and $T^{\prime }$ of a given target T and organ O on the cranio‐caudal axis. The OZH is a one‐dimensional function dependent on the distance r from the PTV and can be calculated by Equation (2).

(2) $$\mathrm{OZH}\left(r\right)=\frac{∥\left\{p\in O^{\prime }|d\left(p,T^{\prime }\right)\leq r\right\}{∥}_{L}}{∥O^{\prime }{∥}_{L}}$$

Here, $d(p,T^{\prime })$ represents the Euclidian distance between a point p within the orthogonal projections of $O^{\prime }$ of the organ and the projection of the boundary of the target. The norm ${∥x∥}_{L}$ is defined, as the maximal distance between two points of the objective x on the cranio‐caudal axis. Since the length of the numerator, associated with a fraction of $O^{\prime }$ is always smaller or equal to the length of the entire organ $O^{\prime }$ in the denominator, the OZH yields a percentage of the OAR's length. In simpler terms, the OZH quantifies the percentage of the OAR's projected length that intersects with a uniformly expanded or contracted target's projection.

The calculation of the OZH involves two main steps: target expansion and target contraction. In the first step, the target is uniformly expanded in all directions. The overlap length between the expanded target and OAR is calculated. This expansion is repeated until the target fully encompasses the OAR, with the overlap length being the OAR's length. In the second step, the target is uniformly contracted in all directions until there is no overlap between the contracted target and the OAR. Again, during each step, the overlap length between the contracted target and OAR is calculated. The resulting curve from the combination of target expansion and contraction is the OZH, which describes the relationship between the target and the OAR within the specified distance in cranio‐caudal direction.

### 2.3 Properties of OVH and OZH

**FIGURE 1:**
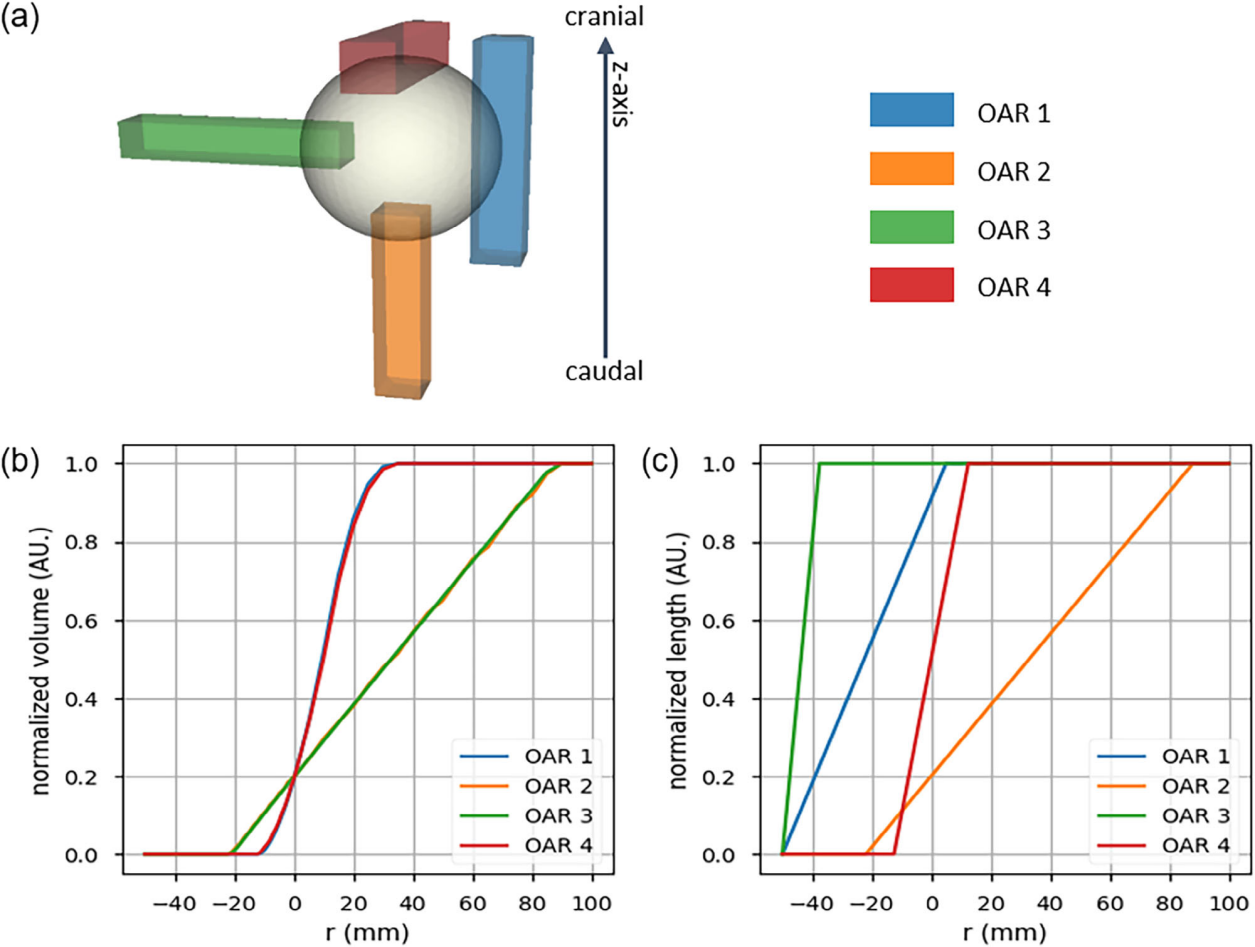
A simplified example configuration of four OARs and one target according to OVH and OZH curves. (a) 3D shapes of target and OARs. The OARs have identical shapes to indicate characteristics of OVH and OZH. (b) OVH and (c) OZH of the shown 3D shapes. OVH curves for OAR1 and OAR4 as well as for OAR2 and OAR3 are exactly overlapping. OAR, organs‐at‐risk; OVH, overlap volume histogram; OZH, overlap‐*z*‐histogram.

The combination of OVH and OZH offers a method to describe the spatial distribution of a target and the relevant OARs. A simplified example configuration of four OARs and one target is illustrated in Figure 1a. The target is simplified as a sphere with a radius of 5 cm. The four equivalent‐sized boxes represent OARs with dimensions of 2.5 × 2.5 × 11 cm^3^. Due to operations, including rotations and shifts, the OARs exhibit distinct spatial relationships with the target. The OVH and OZH curves for all OARs are depicted in Figure 1b,c respectively. The OVH curves in Figure 1b reveal that OAR1 and OAR4 as well as OAR2 and OAR3 have a similar volume overlap relationship to the target, resulting in the same OVH curves, respectively.

This behavior comes from a rotational symmetry of the OVH. The OVH curves also demonstrate that OAR2 and OAR3 penetrate much deeper into the target compared to OAR1 and OAR4. The penetration lengths of OAR1 and OAR4 within the target are each 1.25 cm. OAR2 and OAR3 are placed deeper in the target, with a length of 2.2 cm. Even if they have a different penetration depth, their overlap without enlargement or contraction is about the same at OVH(0)≈20%. It is noteworthy that this metric of overlap volume is a single point, which is located at the zero crossing in the OVH curve and is always included in the OVH curve, even if there is no overlap between the target and OVH. Furthermore, the slope of the OVH curve reveals information about the shape and position of the OAR in relation to the target. As in this example all OARs have the same shape, the positioning has an influence on the rate at which the target encompasses the OAR. For instance, to cover 80% of the volume of OAR1, the target must expand by 1.8 cm, while covering the same percentage of OAR2 requires a target expansion of 6.6 cm.

To detect further spatial differences between the four OARs, the OZH curves displayed in Figure 1c can be examined. Compared to the OVH, with only two different curves for four OARs, the OZHs for each OAR are different to each other.

Through the application of target contraction, the OZH curves highlight that OAR3 has a higher cranio‐caudal overlap than all other OARs. Even with a contraction of 3.75 cm the target and OAR3 share the same transverse planes, meaning that OAR3 is much thinner than the target in cranio‐caudal direction. OAR2 on the other hand, which has the exact same OVH, has only a small length overlap in OZH. After a contraction of 1.25 cm OAR2 has no more OZH overlap. The point OVH(R=0) on the OZH curve reveals the overlap on cranio‐caudal axis without contraction or expansion. OAR4, for example, is 50 % at the target level, while OAR1 has an overlap of almost 90 %.

Through this simple arrangement of the OARs around the target and the OVH and OZH curves obtained, assumptions about the potential sparing of the OAR and thus about the dose within the OARs can be made. The OVH curves indicate that the non‐overlapping portions of OAR2 and OAR3 can be spared more easily than those of OAR1 and OAR 4. Due to the information of OZH, and the condition of coplanar VMAT planning the non‐overlapping part of OAR2 can be spared easily, estimating a lower dose than for OAR3. The same is valid for OAR4, which has a lower non‐overlapping portion in OZH than OAR1. Hence, the combined information of OVH and OZH is an important component of DVH prediction.

### 2.4 Creating a database for DVH prediction

For the creation of the knowledge‐based DVH prediction model for lung tumors, a basis of 106 lung tumor patients (44 female, 62 male) with varying PTV locations was used. All patients were treated coplanar with VMAT within the routine workflow. Further restrictions concern a total dose of 5040 cGy, a fraction pattern of 28 fractions, and a photon beam (flattened beam) energy of 6 MV. The size of the PTV ranged from 85 $cm^{3}$ to 1672 $cm^{3}$ (average: 601 $cm^{3}$). The patients were treated between 2019 and 2025, the treatments were planned by 15 different physicists, using the treatment planning system (TPS) RayStation (RaySearch Laboratories, Stockholm, Sweden). A mean coverage of 95.6 was achieved for all patients within the cohort. At the time of treatment, the patients were between 42 and 84 years old (mean: 66.0). Details are listed in Table 1.

**TABLE 1:** Patient collective (N = 1) details. Lung tumor patients were treated normo‐fractionated with 180 cGy/fx in 28 fractions and a beam energy of 6 MV.

	*N*	(%)
**Age**		
<50	3	2.83%
50–59	22	20.75%
60–69	43	40.57%
70–79	31	29.25%
>79	7	6.60%
**Sex**		
Female	44	41.51%
Male	62	58.49%
**Coverage**		
(90,91)	4	3.77%
(91,92)	8	7.55%
(92,93)	7	6.60%
(93,94)	6	5.66%
(94,95)	8	7.55%
(95,96)	25	23.58%
(96,97)	20	18.87%
(97,98)	11	10.38%
(98,99)	13	12.26%
(99,100)	4	3.77%

For each patient plan, the approved RT Structure Set, RT‐dose, and RT plan were exported from the TPS. This data was collected in an independent database. RT Structure Set and RT‐dose were combined and utilized for DVH calculation needed for the validation of the prediction, while the RT Structure Set on its own was processed for OVH and OZH calculation. The mentioned DVH, OVH, and OZH curves were determined for the heart, spinal cord, esophagus, total lung, and in the case of female patients for the left and right breast.

### 2.5 DVH prediction algorithms

In order to predict DVH curves of OARs using OVH and OZH a correlation between the combined OVH‐OZH information and the DVH curves has to be assumed. Simplified, similar OVH and OZH curves of two patient plans for the same OAR should result in similar DVH curves. Hence, a metric of similarity between curves was defined by either their absolute difference in area, see Equation 3 and 4, or their absolute difference in one point, for example at r=0 on their curve, see Equation 5 and 6.

(3) $$\Delta V_{\mathrm{OVH}}=\int_{a}^{b}\left|\mathrm{OVH}\left(r\right)-\mathrm{OV}H_{\mathrm{ref}}\left(r\right)\right|\mathrm{dr}\;$$

In Equation 3, $\mathrm{OV}H_{\mathrm{ref}}\left(r\right)$ represents the OVH curve of a reference patient for whom comparable OVH curves are being searched, while OVH(r) is an OVH curve of another patient in the database. The smaller the difference in area between $\mathrm{OV}H_{\mathrm{ref}}\left(r\right)$ and OVH(r) the more similar the curves are. The same relation is valid when looking at the area difference of OZH curves, in Equation 4.

(4) $$\Delta V_{\mathrm{OZH}}=\int_{a}^{b}\left|\mathrm{OZH}\left(r\right)-\mathrm{OZ}H_{\mathrm{ref}}\left(r\right)\right|\mathrm{dr}$$

For both Equations (3 and 4), the lower and upper bounds of integration depend on the tumor location and the OARs. For lung tumors and OARs a lower bound of a=−100mm and an upper bound of b=300mm was set. This ensured that a complete OVH and OZH between 0 and 1 could be analyzed for every OAR in the database.

For lengthy OARs and the esophagus in particular, it is not sufficient to consider only the entire OVH or OZH curves but also the difference at individual points on the curves, namely at OVH(*r* = 0) or OZH(*r* = 0) is relevant as defined in Equations 5 and 6:

(5) $$\Delta L_{\mathrm{OVH}}=\left|\mathrm{OVH}\left(0\right)-\mathrm{OV}H_{\mathrm{ref}}\left(0\right)\right|$$

(6) $$\Delta L_{\mathrm{OZH}}=\left|\mathrm{OZH}\left(0\right)-\mathrm{OZ}H_{\mathrm{ref}}\left(0\right)\right|$$

To predict a DVH for an OAR of a reference patient, plans with the smallest possible $\Delta V_{\mathrm{OVH}}$ and $\Delta V_{\mathrm{OZH}}$, $\Delta L_{\mathrm{OVH}}$ or $\Delta L_{\mathrm{OZH}}$ had to be found in the database. The values were calculated for the OAR for each individual patient. The selection and order of $\Delta V_{\mathrm{OVH}}$, $\Delta V_{\mathrm{OZH}}$, $\Delta L_{\mathrm{OVH}}$ and $\Delta L_{\mathrm{OZH}}$ has a major influence on the quality of the prediction. Hence, for each OAR different combinations were tested and analyzed to achieve the best forecast results. The methods focused either more on OVH, OZH or on both equally. Three primary approaches have been developed and are detailed below. In Method 1, named OVH‐only, only $\Delta V_{\mathrm{OVH}}$ was used to identify *n* plans with similar spatial relationship between their OAR and their PTV. Here, *n* represents the desired number of predicted DVHs. A database query was performed to determine the plans with the *n* smallest Δ*V*_OVH_ values for the given OAR. The DVHs associated with these *n* plans then formed the prediction. In Method 2, named OVH‐OZH, $\Delta V_{\mathrm{OVH}}$ and $\Delta V_{\mathrm{OZH}}$ information was combined. For the query Δ*V*_OVH_ and Δ*V*_OZH_ were summed and the *n* plans with the lowest Δ*V*_OVH_ +Δ*V*_OZH_ were determined. The DVHs associated with these plans again formed the DVH prediction. Method 3 was specialized for the prediction of esophagus DVHs and is in the following called esophagus‐method. It is based on three‐step filtering: First, identifying the *n* plans with smallest Δ*L*_OVH_. Secondly, searching for *m* out of *n* patient plans with smallest Δ*V*_OZH_ and thirdly identifying *l* out of *m* plans with lowest Δ*L*_OZH_. As with the other two methods, the DVHs of these *m* plans formed the DVH forecast. By repeating the evaluation shown below for different *n*, an optimum value of *n* = 4 was determined for Method 1 and 2. At this value, the best prediction results were achieved with a simultaneously high number of predicted curves. In that way, for the third method the combination of *n *= 9, *m *= 3, and *l *= 2 was optimal.

### 2.6 Validating the DVH prediction

To compare and to become handy with the prediction capabilities of the OVH‐only, OVH‐OZH methods, a preliminary study was made for heart and lung for one patient. The DVH predictions for both OARs were analyzed for a randomly selected patient in the database. To highlight the limitations of the OVH‐only algorithm, we compared the CT images of a randomly selected patient with images from a patient predicted by the algorithm for the heart. Even though both had similar OVH curves, the predicted patient's heart showed a significantly different DVH curve.

Furthermore, as a measure for the quality of prediction, the coefficients of determination ($R^{2}$) of the linear fit were calculated. Therefore, the dose of one predicted DVH was plotted against the archived dose of the DVH actually obtained for the patient concerned. On this curve, a linear fit was applied. The $R^{2}$ of the linear regression model would be close to 1 for a good fit. To validate the prediction capabilities of the previously described methods for a larger patient cohort, the dataset of 106 plans was randomly divided into training and test sets in an 80% to 20% ratio. Specifically, the training set contained 84 patients, while the test set included 22 patients. The clinically achieved DVHs of these 22 test patients were compared to the DVHs predicted by the OVH‐only, OVH‐OZH, and esophagus prediction methods, each trained on the 84 training patients. Differences in DVH prediction accuracy were then analyzed for each OAR separately. To assess whether there is a significant difference in the $R^{2}$ between OVH‐only and OVH‐OZH for the total lung, a Wilcoxon signed‐rank test was applied.^21^ In order to classify the results, the best possible $R^{2}$ that can be achieved based on our database was determined, by searching for the four predicted DVH curves with the highest $R^{2}$ for each achieved DVH curve. Additionally, the DVH metrics displayed in Table 2 were employed. The discrepancy between predicted and achieved DVH metrics was analyzed statistically, using mean values and the root mean square error (RMSE), see Equation 7.

(7) $$\mathrm{RMSE}\;=\sqrt{\frac{\sum {\left(\mathrm{Predicted}-\mathrm{Achieved}\right)}^{2}}{N\mathrm{plans}}}$$

**TABLE 2:** All DVH metrics for lung VMAT plans, which are analyzed.

OAR	Metric
Total lung	Mean (Gy)
	$V_{20\;\mathrm{Gy}}$ (%)
Heart	Mean (Gy)
Esophagus	$D_{0.5\;\mathrm{cc}}$ (Gy)
Spinal cord	$D_{0.01\;\mathrm{cc}}$ (Gy)
Left breast	Mean (Gy)
Right brest	Mean (Gy)

Abbreviations: DVH, dose volume histogram; OAR, organs‐at‐risk; VMAT, volumetric‐modulated arc therapy.

The area under the curve (AUC) was considered as the last measure of predictive quality. The AUC was calculated for both the achieved and the predicted DVHs. The difference was calculated for each individual prediction and visualized using box plots.

## 3 RESULTS

### 3.1 Exemplary DVH prediction for one patient

**FIGURE 2:**
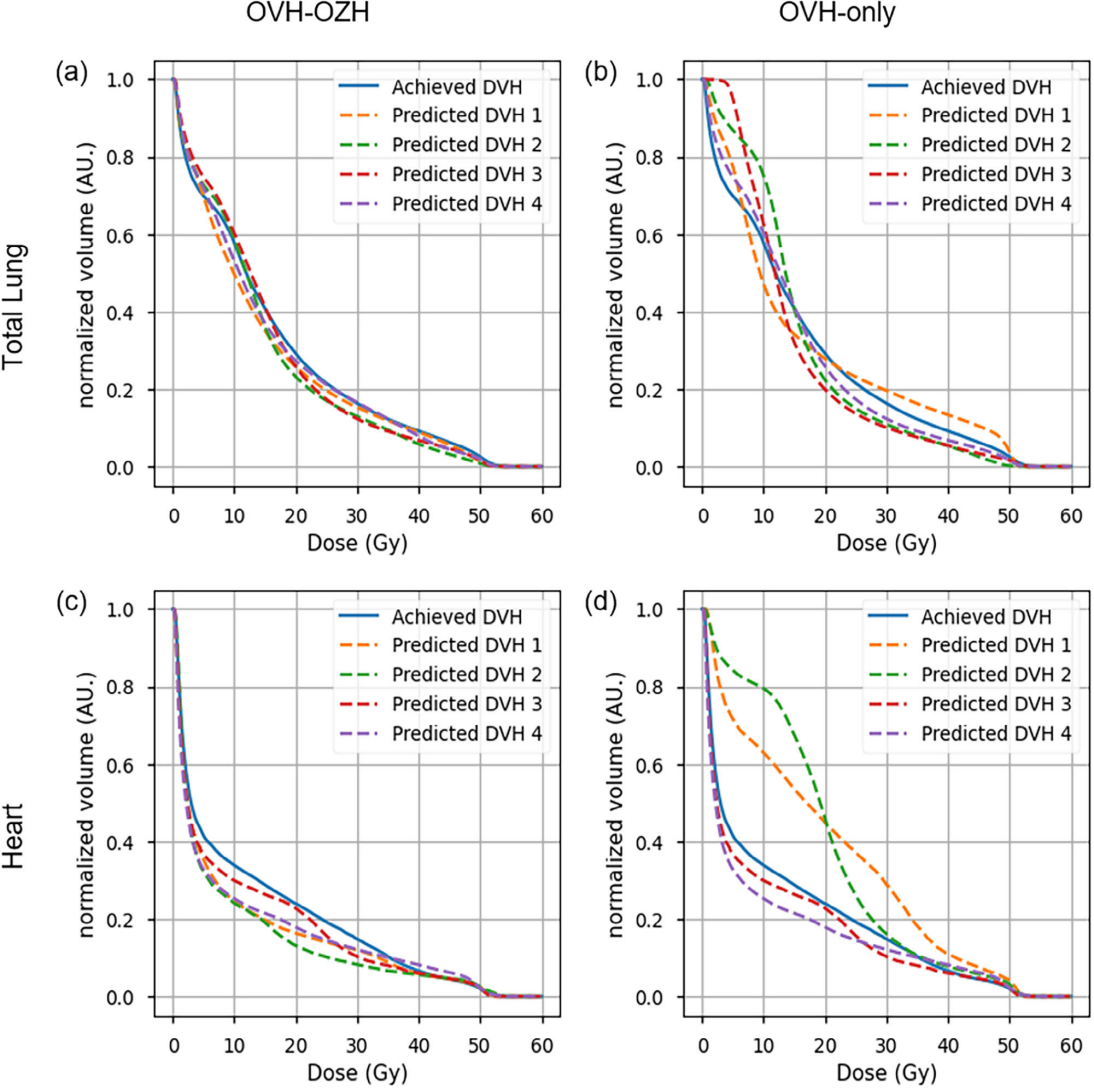
Predicted and for example, the patient achieved DVHs for total lung and heart and two prediction algorithms. Total lung DVHs for (a) OVH‐OZH and (b) OVH‐only prediction algorithms. Predicted and achieved heart DVHs for (c) OVH‐OZH and (d) OVH‐only prediction algorithms. DVH, dose volume histogram; OAR, organs‐at‐risk; OVH, overlap volume histogram; OZH, overlap‐*z*‐histogram

Using the DVH prediction algorithms, Figure 2 presents the DVH forecast for the total lung and heart of a from the test data set randomly selected example patients each in comparison to the ones of the clinical plan. For both, heart and total lung, the OVH‐OZH algorithms resulted in a better alignment with the DVH curves achieved by the planners. For the heart, it is evident for the OVH‐OZH method that all predicted DVHs were slightly lower than the achieved DVHs.

**FIGURE 3:**
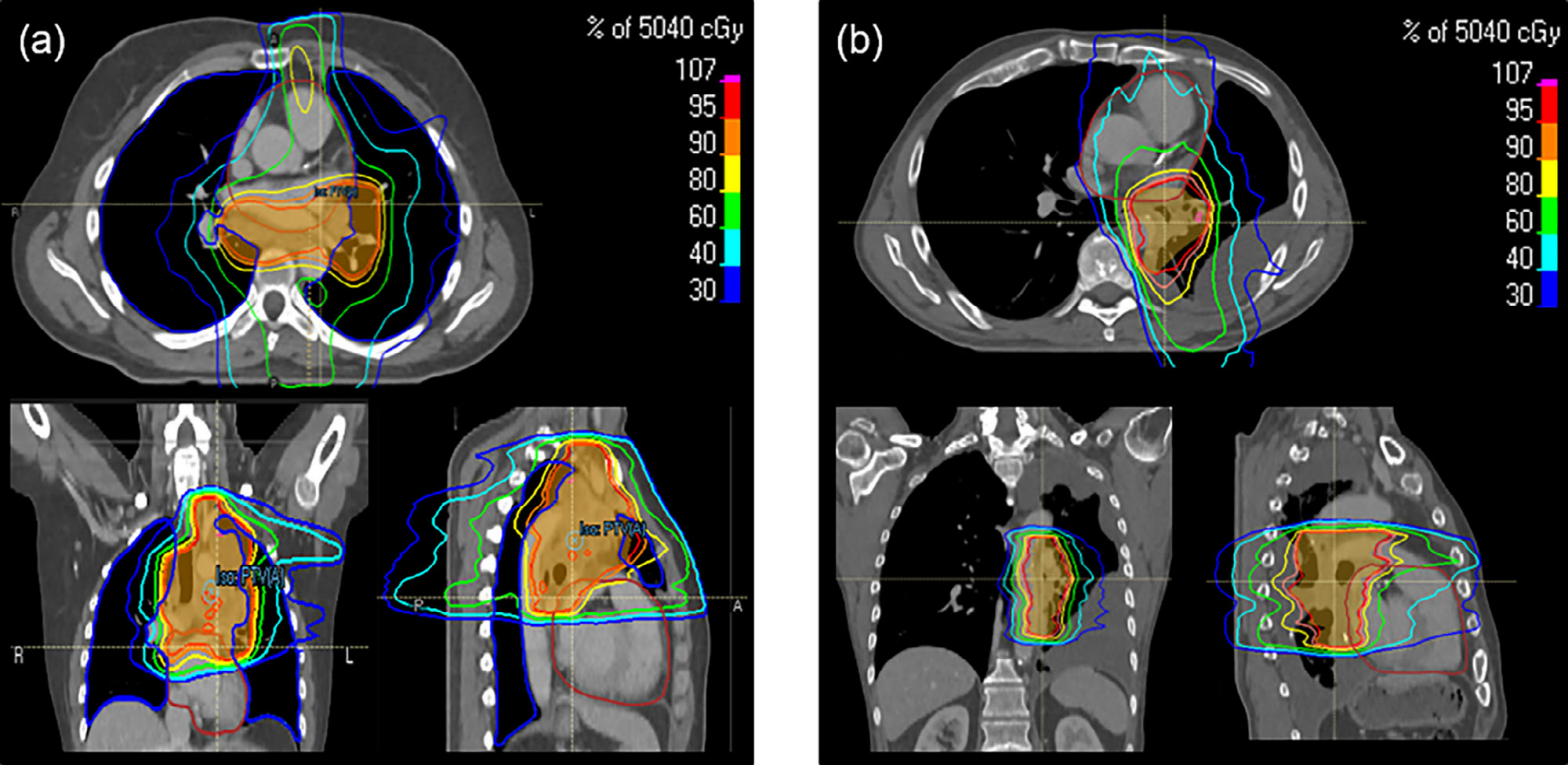
Transverse, coronal, and sagittal plane of CT‐images of two patients with the same heart OVH. The displayed dose distribution and the delivered dose in the heart indicate a dependency on the cranio‐caudal overlap between the heart and the PTV. OVH, overlap volume histogram; PTV, planning target volume.

A clear difference in predictive capability between the OVH‐only and OVH‐OZH methods is visible for the heart. To retrace the outliers in the OVH‐only prediction for the heart, Figure 3a,b show the CT images of the example patient and of the patient corresponding to predicted DVH 1 in Figure 2d. As expected and calculated by the OVH‐only algorithm, both patients exhibited an almost identical relative volume overlap between the heart and PTV. However, since the OVH has rotational symmetry, there were significant differences in the cranio‐caudal arrangement of the PTV and the heart in this case, leading to varying degrees of heart sparing due to the coplanarity of the treatment planning.

To illustrate the difference between OVH‐only and OVH‐OZH methods, the differences in prediction capability are summarized by the $R^{2}$ in Table 3. For both the heart and the total lung, the mean of the $R^{2}$ over the four predicted plans for the OVH‐OZH algorithm was larger than for the OVH‐only method and close to 1.

**TABLE 3:** $R^{2}$ comparison between OVH‐OZH and OVH‐only prediction algorithm.

	OVH‐OZH	OVH‐only
	Mean($R^{2}$)	Mean($R^{2})$
Total lung	0.993 ± 0.002	0.971 ± 0.016
Heart	0.972 ± 0.016	0.931 ± 0.060

Abbreviations: OVH, overlap volume histogram; OZH, overlap‐*z*‐histogram.

### 3.2 DVH prediction results on the test dataset

The results of the $R^{2}$ analysis as well as the best possible $R^{2}$ that can be achieved based on our test dataset are shown in Table 4. For both applied prediction methods, lung, and heart exhibited a strong linear correlation between predicted and achieved DVH data points with $R^{2}$ values close to 1. This indicated that the predicted DVH curve nearly overlapped the achieved DVH curve. With a mean $R^{2}$ of 0.975 ± 0.022 the prediction capability for the total lung of Othe VH‐OZH method was slightly better than for the OVH‐only (0.968 ± 0.025) method. The null hypothesis of the Wilcoxon‐Test, that the distribution of $R^{2}$ with OVH‐OZH is the same as the distribution with OVH‐only, was rejected at a confidence level of 5%, with a *p*‐value of 0.003. The highest difference between OVH‐only (0.897 ± 0.036) and OVH‐OZH (0.962 ± 0.036) method was visible for the heart's DVH prediction. For spinal cord, the linear fit $R^{2}$ values were 0.905 ± 0.079 and 0.902 ± 0.081 for OVH‐OZH and OVH‐only, respectively. The best prediction for the esophagus was achieved with the specially designed method (0.913 ± 0.083).

**TABLE 4:** $R^{2}$ comparison between OVH‐OZH, OVH‐only, and esophagus prediction algorithm, analyzed on the training set.

	Best possible	OVH‐OZH	OVH‐only	Esophagus‐method
	Mean(*R* ^2^)	Mean(*R* ^2^)	Mean(*R* ^2^)	Mean(*R* ^2^)
Total lung	0.992 ± 0.004	0.975 ± 0.022	0.968 ± 0.025	
Heart	0.985 ± 0.009	0.962 ± 0.036	0.897 ± 0.087	
Esophagus	0.963 ± 0.026	0.894 ± 0.085	0.912 ± 0.079	0.913 ± 0.083
Spinal cord	0.981 ± 0.017	0.905 ± 0.079	0.902 ± 0.081	
Left breast	0.973 ± 0.021	0.907 ± 0.095	0.897 ± 0.082	
Right breast	0.962 ± 0.093	0.919 ± 0.095	0.900 ± 0.103	

*Note*: The mean was calculated over all four predictions of every test patient.

Abbreviations: OVH, overlap volume histogram; OZH, overlap‐*z*‐histogram.

The differences in the DVH metrics, including their mean and RMSE values across all four predictions for each patient in the test dataset, are presented in Table 5. The OVH‐OZH method substantially reduced the RMSE for the mean heart dose, lowering it from 5.89 Gy with OVH‐only to 1.92 Gy. A similar trend was observed for the total lung, where the RMSE for the mean dose was reduced from 1.59 Gy (OVH‐only) to 1.09 Gy (OVH‐OZH), indicating a minor but noticeable improvement. For lung $V_{20\;Gy}$, both methods yielded nearly identical RMSE values (0.03 %) and mean values less than 0.01 %. In breast mean dose predictions, OVH‐OZH slightly reduced RMSE compared to OVH‐only, from 3.05 Gy to 3.01 Gy for the left breast and from 4.38 Gy to 4.11 Gy for the right breast. Additionally, it improved mean dose prediction by reducing underestimation, with the left breast improving from −0.77 Gy to −0.15 Gy and the right breast from −1.59 Gy to −0.92 Gy. For the $D_{0.5\;\mathrm{cc}}$ of the esophagus, the specialized Esophagus‐method achieved the best prediction accuracy, with an RMSE of 6.26 Gy, compared to 6.75 Gy (OVH‐only) and 7.24 Gy (OVH‐OZH). The $D_{0.01\;\mathrm{cc}}$ of the spinal cord followed a similar trend, where OVH‐OZH resulted in a slightly higher RMSE (5.02 Gy vs. 4.34 Gy for OVH‐only) and a marginally increased mean dose (1.19 Gy vs. 1.00 Gy).

**TABLE 5:** DVH metrics comparison for OVH‐OZH, OVH‐only, and esophagus prediction algorithms for all patients in the training dataset.

		OVH‐OZH		OVH‐only		Esophagus‐method	
OAR		Mean	RMSE	Mean	RMSE	Mean	RMSE
Total lung	Mean (Gy)	−0.11	1.09	−0.11	1.59		
	$V_{20\;\mathrm{Gy}}$ (%)	0.00	0.03	0.01	0.03		
Heart	Mean (Gy)	−0.59	1.92	−0.83	5.89		
Esophagus	$D_{0.5\;\mathrm{cc}}$ (Gy)	1.34	7.24	1.12	6.75	1.12	6.26
Spinal cord	$D_{0.01\;\mathrm{cc}}$ (Gy)	1.19	5.02	1.00	4.34		
Left breast	Mean (Gy)	−0.15	3.01	−0.77	3.05		
Right brest	Mean (Gy)	−0.92	4.11	−1.59	4.38		

*Note*: The mean and RMSE were calculated over all *four predictions of every test patient*.

Abbreviations: DVH, dose volume histogram; OAR, organs‐at‐risk; OVH, overlap volume histogram; OZH, overlap‐*z*‐histogram; RMSE; root mean square error.

**FIGURE 4:**
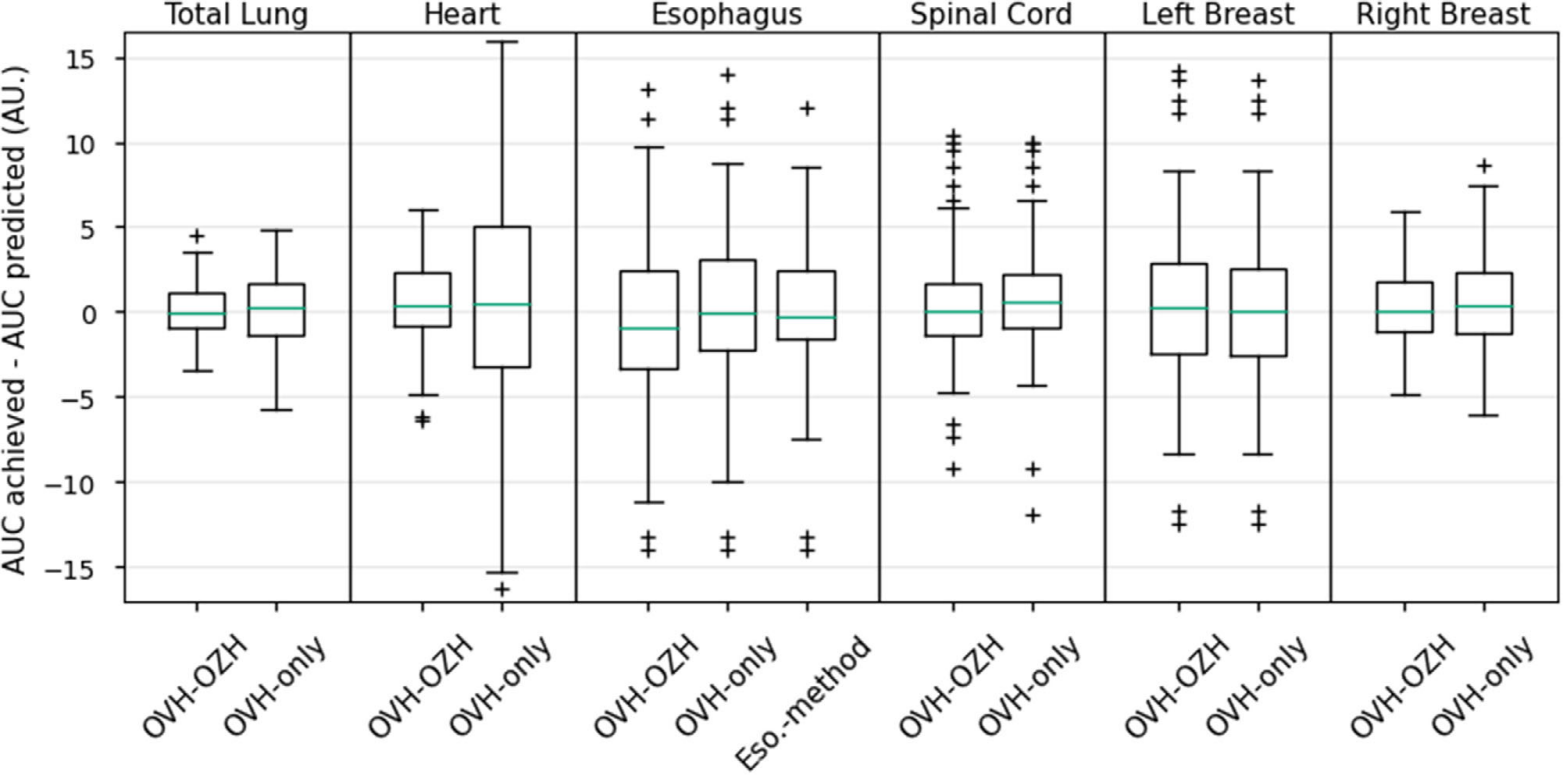
Box plots for the difference between the AUC of the predicted and the achieved DVHs for the OVH‐OZH, OVH‐only, and esophagus prediction algorithms. AUC, area under the curve; DVH, dose volume histogram; OVH, overlap volume histogram; OZH, overlap‐*z*‐histogram.

Figure 4 presents the AUC analysis results. The box plots for OVH‐OZH and OVH‐only showed minimal differences for both breasts and the spinal cord. However, the heart benefited most from incorporating OZH, with fewer outliers, a median closer to zero, and a smaller interquartile range. For the lung, OVH‐OZH outperformed OVH‐only, showing a narrower spread and a median closer to zero. The Esophagus‐method achieved the highest predictive accuracy for the esophagus, with less variability and a median closer to zero, indicating better alignment between predicted and achieved AUC values.

## 4 DISCUSSION

This study aimed to develop a knowledge‐based automated DVH prediction module for OARs of lung tumors, primarily focusing on the impact of incorporating OZH alongside OVH. Therefore, the prediction capabilities of the OVH‐only and OVH‐OZH methods were analyzed.

Both methods demonstrated DVH prediction capabilities for most organs, achieving notable success in total lung. However, the various evaluations also highlighted the limitations of the OVH‐only method and its inferior predictive ability compared to the OVH‐OZH method. Especially the heart benefited from the combination of OVH and OZH. While an initial assessment suggested that OVH predictions might suffice for the esophagus, the study revealed that incorporating OZH had a positive influence on prediction accuracy. This unexpected finding suggests that, like other organs, the esophagus can benefit from a combined OVH‐OZH approach.

Despite the overall success, the spinal cord presented challenges in prediction due to its sensitivity to treatment planning nuances. The planar dependency of spinal cord DVH predictions highlighted the complexity of accurately foreseeing outcomes in this particular organ. The prediction of DVH metrics for spinal cord and esophagus appeared to be subject to greater scatter, not only because of the different emphasis of the planner but also due to the consideration of near‐maximum doses rather than mean doses. Even though the planning effort to minimize the spinal cord dose below for example, 45 Gy is minimal, it seemed to be a notorious structure for inconsistent planning. While an initial assessment suggested that OVH predictions might suffice for the esophagus, the study revealed that incorporating OZH had a positive influence on prediction accuracy. The prediction of DVH metrics for the spinal cord and esophagus showed greater variability. This variability arised not only from differences in the planner's emphasis but also from the focus on near‐maximum doses rather than mean doses. Remarkably, a reliable prediction was also achieved for the female breast, even though it is rarely considered in daily planning, as the focus is usually on higher‐risk organs like the heart and lungs.

Compared to other knowledge‐based DVH prediction methods, the methods shown here performed slightly worse at first glance, but this is mainly due to the fact that they were primarily evaluated for other tumor regions, such as the head and neck.^5^, ^14^, ^16^, ^22^ Due to smaller differences in size and position of the OAR and the PTV, there are also smaller DVH differences. However, compared to other knowledge‐based prediction methods that were evaluated for lung cancer, a clear benefit in DVH prediction can be recognized due to the OZH. While Polizzi et al.^12^ concluded in 2022, that despite exact OVH matching, the DVH of the heart is subject to large fluctuations and thus prediction is very poor, the methods shown in this study can explain these fluctuations and furthermore enable DVH for the heart using OVH and its extension of OZH.

In this study, the number of plans to be predicted with similar DVH was set to either 4 or 2, depending on the method and OAR. As described above, the number was varied and the prediction quality was compared. Varying this number naturally leads to slightly different results in the prediction quality, even if this does not change the absolute trend of better prediction by the OVH‐OZH method. For a larger database, with a larger number of plans with a similar spatial distribution, it is recommended to increase the number of predicted plans. This allows outlier plans to be recognized even better while maintaining the prediction quality.

Due to the relatively small database, the random splitting of the data into training and test sets for evaluation lead to substantial variations in the results, depending on which patients were included in each subset. In some of these random splits, especially for the lung, even better results were achieved using the OVH‐OZH method. Nevertheless, the fundamental statement remains valid that the OVH‐OZH method provided added value, particularly for OARs showing a strong cranio‐caudal overlap with the PTV, such as the lungs and the breast, regardless of how the data were randomized.

Especially for the esophagus, but also for the heart and the right and left breast, there were plans in the database that could not be predicted or could only be predicted very poorly, as there were no matching OVHs and/or OZHs. For the esophagus, for instance, there were only three plans without overlap between PTV and esophagus. To address these challenges and improve the overall prediction quality, one option could be enlarging the database to increase diversity and improve the matching of OZH and OVH patterns. Additionally, strategic exclusion of plans with inferior dose sparing, based on a comprehensive dataset of high‐quality plans, was suggested to refine DVH predictions. Developing a scoring system and engaging medical expertise are necessary steps for this exclusion. Comparative studies have shown mixed results regarding the impact of removing suboptimal plans from the database. Some suggest that the database is self‐cleaning and that removing outliers does not significantly affect performance, while others indicate that a cleaned‐up knowledge‐based prediction model can improve the accuracy of estimated DVHs, particularly in high‐dose regions.^23^ The findings suggest that while a cleaned‐up model can enhance prediction accuracy, its effectiveness for new patients remains to be fully determined. Another way to generate a highly consistent OVH database and thereby improve DVH prediction would be to clean the database using automatically optimized multi‐criteria treatment plans as evaluated by Wang et al.^24^ Non‐coplanar treatment planning can positively influence the dose sparing of OARs around the PTV depending on their location. To integrate non‐coplanar plans into the database and predict their DVHs, the prediction technique needs to be adjusted to take into account parameters such as couch rotation and gantry start and stop angles. Non‐coplanar plans can distort the OZH values, leading to overestimation of doses for organs such as the heart. For the lungs and elongated organs such as the spinal cord and esophagus, there are only minimal differences between coplanar and non‐coplanar plans in terms of dose effect. To avoid strong over‐ or underestimation of doses in the heart, introducing additional metrics, such as binning by couch and gantry rotation angles, could help. Implementing this binning requires a larger database, but initial trials were promising about the potential for improved DVH prediction quality for non‐coplanar lung cancer VMAT plans using a combination of OVH, OZH, and angle binning. An example DVH prediction for one patient shows a clear application of the DVH prediction method. As described, the DVH curves predicted for the heart using the OVH‐OZH method were all lower than the DVH achieved by the planner. The plan was reviewed after the DVH prediction and it was determined that further heart sparing may be possible without much effort. Using this DVH prediction and analysis of the 4 predicted DVHs, it was possible to identify a plan that was not yet fully optimized in clinical practice, highlighting the value of the OVH‐OZH prediction method shown here.

## 5 CONCLUSION

The quality of a VMAT treatment plan depends not only on patient specific factors including the type and stage of cancer, the location, and on size of PTV and OARs, but also on the planner's level of experience, preferences, and the amount of time the planner is able to invest in the plan. Particularly in the case of lung tumors, deciding whether it is possible or worth spending more time to further spare surrounding organs is difficult. To address this issue, a knowledge‐based automated DVH prediction module for lung tumors was built. Using OVH and OZH simultaneously this method provided an effective quality control mechanism for evaluating the DVHs of the OARs. Implementing this method in the clinical workflow of treatment planning will contribute to advance the quality of VMAT plans and, furthermore, help to avoid the unnecessary overdosing of OARs. Running the DVH prediction before planning, will shorten the planning time, especially for inexperienced planners. The database and workflow build a basis, which is expandable to other tumor indications and applications such as spatial information dependent scoring and automated treatment planning.

## AUTHOR CONTRIBUTIONS

All authors contributed significantly to the performed work and approved the final version of the manuscript to be published. *Conceptualization, Methodology, Software, Writing—Original Draft*: Johann Brand. *Conceptualization, Supervision, Writing—Review & Editing, Visualization*: Juliane Szkitsak. *Resources, Review*: Oliver J. Ott. *Supervision, Writing—Review & Editing*: Christoph Bert. *Conceptualization, Supervision, Writing—Review & Editing*: Stefan Speer.

## CONFLICT OF INTEREST STATEMENT

The authors declare no conflicts of interest.

## Data Availability

Data are provided by the corresponding author upon reasonable request
